# Effect of high-frequency electric field on the tissue sticking of minimally invasive electrosurgical devices

**DOI:** 10.1098/rsos.180125

**Published:** 2018-07-11

**Authors:** Liang Zheng, Jianfei Wan, Yunjiang Long, Helin Fu, Jing Zheng, Zhongrong Zhou

**Affiliations:** 1 Tribology Research Institute, Key Laboratory of Advanced Technologies of Materials, Ministry of Education of China, Southwest Jiaotong University, Chengdu 610031, People's Republic of China; 2 School of Life Science and Engineering, Southwest Jiaotong University, Chengdu 610031, People's Republic of China

**Keywords:** minimally invasive electrosurgical device, high-frequency electric field, electrode surface, tissue sticking, interfacial binding strength

## Abstract

Generally minimally invasive surgery is performed using an endoscope and other instruments including electrosurgical units (ESUs), and the adhesion of tissue to electrodes is a major concern. The mechanism governing this tissue sticking, especially the influence of high-frequency electric field, is still unclear. In this study, the effect of high-frequency electric field on the tissue sticking upon electrodes was investigated. The electrosurgical cutting test was performed on *ex vivo* fresh porcine liver under blend mode using a monopolar ESU. A heat-adherence test without electric field was used as a control. For the control group, the electrode was heated and maintained at a certain temperature and directly in contact with porcine liver. Both sticking tissues obtained from these two tests are partially carbonized porcine liver tissue, but their microstructure and bonding with electrode are obviously different. The sticking tissue formed just under heat is composed of biggish nanoparticles of different sizes which are loosely aggregated and has a weak bonding with the electrode, while the sticking tissue from the electrosurgical cutting test consists of tightly packed fine nanoparticles of equable size as a result of thermo-electric coupling and has a strong bonding with the electrode. Obviously, high-frequency electric field plays an extremely important role in the formation of the sticking tissue. It is the thermo-electric coupling that underlies the function of minimally invasive electrosurgical devices, and the effect of high-frequency electric field cannot be ignored in the tissue sticking study and anti-sticking strategies.

## 1. Introduction

Minimally invasive surgery (MIS) has the advantages such as reduced pain, less tissue injury, quicker return of oral intake, shorter hospitalization and improved cosmetic appearance when compared with open surgery [1,2]. Hence, MIS has taken the place of many traditional open surgical approaches in a number of disciplines over the past two decades. MIS is generally performed using an endoscope and other instruments. As an apparatus for performing incision and coagulation simultaneously, electrosurgical unit (ESU) is one of the widely used endoscopic tools in MIS [3]. The basic components of an ESU include a radio-frequency generator and two electrodes. Depending on whether the two electrodes are separated or not, ESUs fall into two categories, monopolar and bipolar. The monopolar ESU mainly consists of generator, active electrode and dispersive electrode, while the bipolar ESU is designed with two active electrodes positioned together. When the electrode of an ESU approaches targeted tissue during surgical operation, high-frequency current is conducted to the tissue to cause the oscillation of charged intracellular substances and thus generate heat [4]. The thermal effect not only leads to the tissue being incised, ablated and dissected, but also promotes haemostasis. Compared with traditional scalpels, using ESUs significantly reduces the amount of blood loss and separates tissues more rapidly [5,6].

Although ESUs can perform incision and coagulation simultaneously through thermal effect, the resulting smoke and tissue adhesion to electrodes from electrosurgery have troubled surgeons since its first application in clinic [4,7–10]. The occurrence of tissue sticking increases the electrical resistance of electrodes and hinders the energy delivery to targeted tissue, reducing operation accuracy and prolonging surgery. The sticking tissue is very difficult to be cleaned off from the electrode [7]. Thus, surgeons have to periodically suspend their operation and take out the electrode to clean the sticking tissue, which might result in unnecessary bleeding. The sticking problem is already one of the critical challenges that affect the application of minimally invasive electrosurgical devices [11,12], and it has been paid more and more attention in the past decade.

Some solutions have been proposed and used to prevent tissues from adhering to electrodes, such as cold saline irrigation, anti-sticking coatings and surface textures, and thermal management system (TMS) [13–19]. Cold saline irrigation produces steam bubbles upon electrodes which act as a protective layer to prevent the adherence of tissues to electrodes [16]. Almost all the anti-sticking coatings and surface textures on electrodes are used to increase the hydrophobic capacity of electrode surface so as to decrease the tissue adhesion with the electrode [14,17,19]. TMS is used to maintain a cool electrode by conducting heat from the hot electrode to a heat sink and then reduce tissue carbonization and sticking [15,18]. However, these solutions only alleviate tissue sticking to a certain degree, but cannot settle this matter finally. It should be noted that almost all the current anti-sticking approaches are proposed based on the premise that tissue sticking during electrosurgery occurs by the heated tissues adhering to the hot electrode and then suffering carbonization [20–22], while the effect of high-frequency electric field is ignored. In fact, both thermal field and high-frequency electric field are there during electrosurgery. However, up to now, almost no efforts have been made to investigate the role of high-frequency electric field in tissue sticking.

In this paper, the tissue sticking upon 304 stainless steel active electrode during electrosurgery was investigated using a monopolar ESU. For comparison, a heat-adherence test without the influence of electric field was designed by heating and maintaining an electrode at a certain temperature and then putting it in contact with porcine liver. Particular attention was paid to the microstructure of sticking tissue and its bonding with the electrode. The target of this study was to reveal the effect of high-frequency electric field on the tissue sticking.

## 2. Material and methods

### 2.1. Sample preparation

*Ex vivo* fresh porcine liver was used to prepare tissue samples due to its rich capillary blood distribution and adequate blood supply. All the porcine livers were collected from male pigs in a local abattoir, preserved in an icebox and delivered to the laboratory. Porcine liver chunks with a size of about 80 × 50 × 20 mm were used for the electrosurgical cutting test, while porcine liver slices with a size of about 25 × 10 × 0.5 mm were used to do the control test. The tissue sample preparation was completed at a room temperature of 20 ± 3°C and relative humidity of 60 ± 5% within 2 h post-mortem so as to avoid tissue dehydration.

Standard blade-type 304 stainless steel monopolar active electrodes (HUAWEI Medical Instrument Co., Ltd, China) were used for all the tests. To avoid the influence of surface textures resulting from manufacture process on test results, all the electrode samples were polished slightly, and the average roughness *R*_a_ was controlled at about 0.10 µm. All electrodes were treated with ultrasonic cleaning in acetone before testing.

### 2.2. Electrosurgical cutting tests

**Figure 1.**
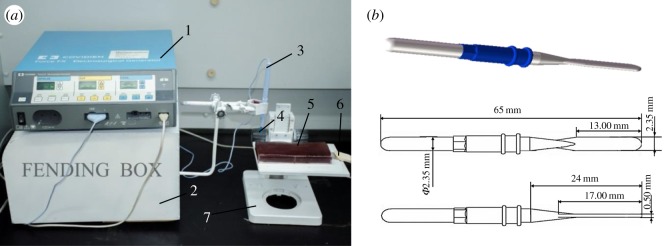
Electrosurgical cutting experimental set-up and electrode sample. (*a*) Electrosurgical cutting experimental set-up, 1—ESU, 2—feeding device, 3—active electrode pen, 4—active electrode, 5—porcine liver, 6—dispersive electrode, 7—locating device. (*b*) Standard blade-type 304 stainless steel monopolar active electrode.

Electrosurgical cutting tests were conducted under the blend mode using a monopolar ESU (Force FX-8CS, Conviden, USA). To avoid the deviation of test results caused by manual operation, all the electrosurgical cutting tests were carried out using a self-designed auxiliary device which mainly consists of a feeding part and a lifting part to precisely control cutting speed and depth, as shown in figure 1. All the tests were finished under the cutting power of 50 W, the cutting speed of 8 mm s^−1^ and the incision depth of 10 mm. To form apparent sticking tissue upon active electrode, a total cutting length of 320 mm and effective cutting time of 40 s were used for each cutting test. To better simulate the real cutting condition of electrosurgery, the choice of these parameters was based on clinical experience.

### 2.3. Heat-adherence test as the control

**Figure 2.**
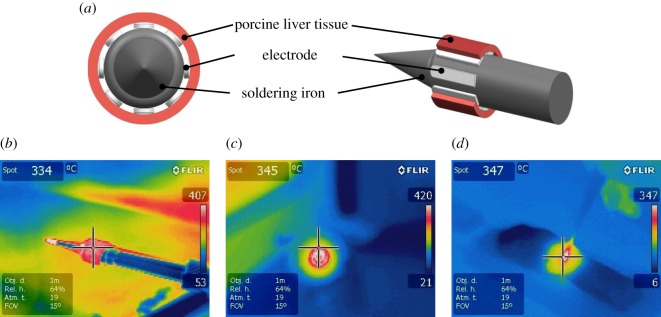
Schematic diagrams of heat-adherence test and thermographs. (*a*) Schema of heat-adherence test. (*b*) Thermograph of heat-adherence test, side view. (*c*) Thermograph of heat-adherence test, front view. (*d*) Thermograph of electrosurgical cutting.

To reveal whether the high-frequency electric field has an influence on the adhesion of tissue to electrodes or not, a heat-adherence test without electric field was designed as the control. For the control group, the front end of electrode, about 10 mm long, was scissored and fixed onto a soldering iron with temperature control, as shown in figure 2. It was firstly heated and maintained at 350°C, and then directly in contact with porcine liver slice. The heat-adherence test lasted about 40 s. The temperature distribution in the porcine liver tissue around the electrode was measured using an infrared thermal imaging camera and then controlled to be approximately the same for the control and experimental groups. The tissue temperature near the electrode was measured to be about 345°C for the control (figure 2*c*) and 347°C for the electrosurgical cutting group (figure 2*d*).

### 2.4. Binding strength measurements

**Figure 3.**
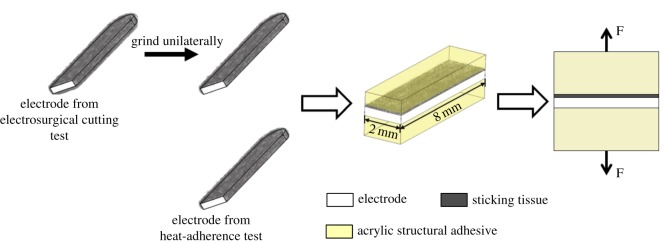
Schematic representation of tensile test sample preparation.

The binding strength of sticking tissue on the electrode was measured by the vertical tension method on a material testing machine (HY0580, Shanghai Hengyi Testing Machine Co., Ltd, China). After the electrosurgical cutting test, the front end of active electrode covered with sticking tissue, about 10 mm long, was scissored, and ground unilaterally using abrasive paper to remove the sticking tissue on its lower surface, as shown in figure 3. Subsequently, it was embedded with acrylic structural adhesive and then ground to obtain a tensile testing sample. After the heat-adherence test, the electrode was taken off from the soldering iron, embedded and then ground to obtain tensile testing sample, as shown in figure 3. For all the tensile samples, the testing area was 8.0 × 2.0 mm, and the tensile test speed was 0.5 mm min^−1^.

The calculation formulae of binding strength (*σ*) and energy dissipation per unit area (*E_a_*) are as follows:

2.1 $$\sigma =\frac{F_{0}}{A_{0}},\;E_{a}=\frac{E_{0}}{A_{0}},$$

where *F*_0_ is the critical force to pull the sticking tissue off the electrode surface, *A*_0_ is the contact area between the sticking tissue and electrode surface, about 16 mm^2^, and *E*_0_ is the energy dissipation during pulling the sticking tissue off the electrode surface.

### 2.5. Morphology and composition characterization

After testing, the surfaces and cross-sections of electrodes were examined and analysed by various methods including a scanning electronic microscope (SEM) (QUANTA200, FEI Corp., UK), an energy-dispersive X-ray detector (EDX) (PV7760/68 ME, EDAX Corp., USA) and a white light interferometer (WLI) (Rtec-MFT3000, San Jose, USA). Fourier transform infrared spectroscopy (FTIR)(Nicole 6700) was used to analyse the functional groups of sticking tissue. The surfaces of electrodes after tensile testing were observed by an optical microscope (OM)(OLYMPUSB 201, Japan).

The tissue near the incision in porcine liver tissue sample was examined using histotomy. After the electrosurgical cutting test, slides were prepared with tissue slices at the incisional midpoint, perpendicular to the experimental incisions. The samples were stained with haematoxylin and eosin. Digital images of each incision were captured with a digital camera-equipped light microscope.

## 3. Results

### 3.1. Morphology and chemical composition

**Figure 4.**
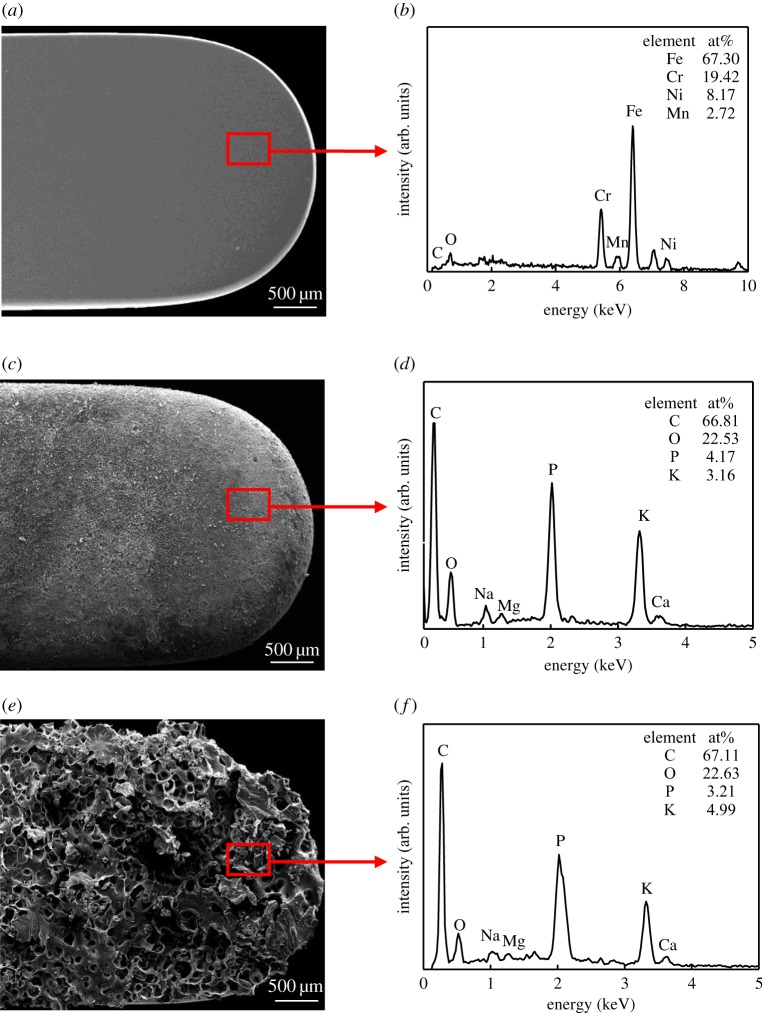
SEM micrographs and EDX spectra of electrode surfaces. (*a*) SEM micrograph of original electrode; (*b*) EDX spectrum of original electrode; (*c*) SEM micrograph after electrosurgical cutting test; (*d*) EDX spectrum after electrosurgical cutting test; (*e*) SEM micrograph after heat-adherence test and (*f*) EDX spectrum after the heat-adherence test.

The surfaces of electrodes were first examined by SEM after the two tests, and figure 4 shows their surface micrographs. The surfaces are covered with a layer of sticking tissue after each of these two tests, but their surface morphologies are obviously different. The electrode is covered with tightly packed small particles after the electrosurgical cutting test, while the covering after the heat-adherence test is porous and honeycomb-like. Further EDX examination demonstrates that both sticking tissues on electrodes have similar chemical composition with major components of carbon (C), oxygen (O), phosphorus (P) and potassium (K) as well as small amounts of sodium (Na), magnesium (Mg) and calcium (Ca), as shown in figure 4*d*,*f*.

**Figure 5.**
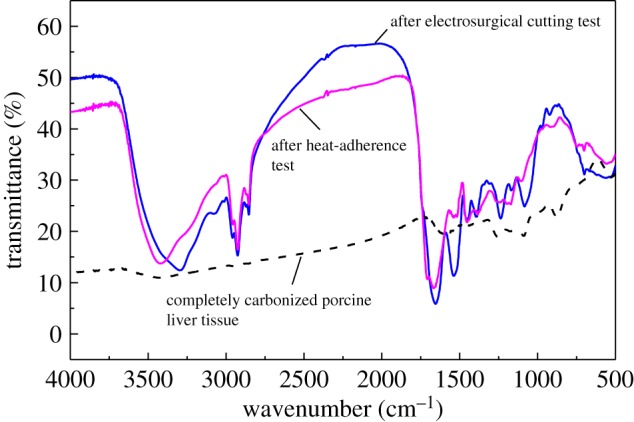
FTIR spectra of sticking tissues on electrode surfaces after the two tests.

Figure 5 illustrates the FTIR spectra of sticking tissues. Completely carbonized porcine liver tissue is used as a control. The sticking tissues have the same functional groups, and they are different from the completely carbonized porcine liver tissue. And no carbonyl (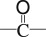) absorption peak appears at 1727–1720 cm^−1^ for the sticking tissues.

**Figure 6.**
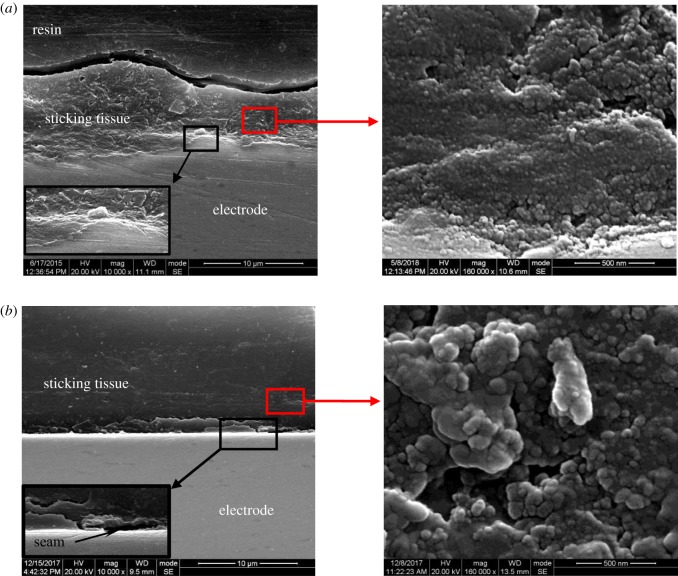
SEM micrographs of electrode cross-sections: (*a*) after the electrosurgical cutting test and (*b*) after the heat-adherence test.

Figure 6 shows the SEM micrographs of the electrode cross-sections. The electrode interface with sticking tissue from the electrosurgical cutting test is scallop-shaped, and there are no obvious seams. The sticking tissue consists of tightly packed nanometre particles of equable size. For the heat-adherence test, the obtained interface is a beeline with obvious seams, and the sticking tissue is porous and composed of biggish nanoparticles of different sizes which are loosely aggregated.

**Figure 7.**
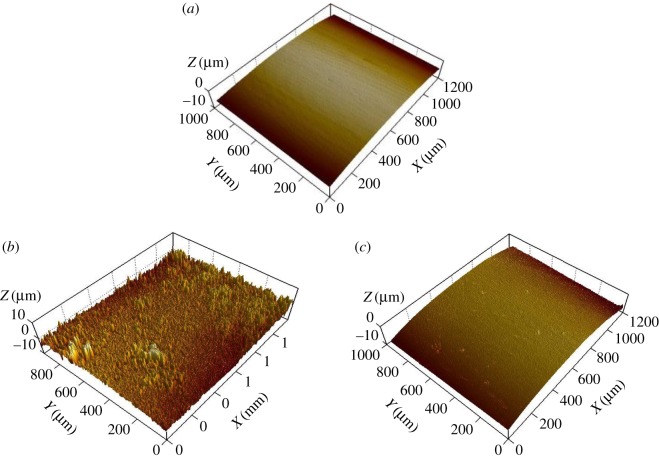
Three-dimensional morphologies of cleaned surfaces of electrodes: (*a*) original electrode; (*b*) after the electrosurgical cutting test and (*c*) after the heat-adherence test.

The electrode surfaces after these two tests were cleaned with acetone-saturated cotton swabs so as to get rid of the adhesions on their surfaces, and their surface morphology was subsequently tested by WLI. As shown in figure 7, the original electrode surface was very even, and it is still even after the heat-adherence test. But, it becomes uneven after the electrosurgical cutting test.

### 3.2. Binding strength

The tensile properties of sticking tissue–electrode interface, such as critical force, binding strength and energy dissipation, are much higher after the electrosurgical cutting test than after the heat-adherence test, as shown in table 1.

**Table 1.** Tensile properties of sticking tissue–electrode interface after the electrosurgical cutting test and the heat-adherence test.

	after electrosurgical cutting test	after heat-adherence test
critical force *F*_0_ (N)	133.4 ± 19.81	8.6 ± 2.3
binding strength *σ* (MPa)	8.34 ± 0.89	0.53 ± 0.14
energy dissipation *E_a_* (10^−3^ J mm^−2^)	5.56 ± 0.57	0.18 ± 0.07

**Figure 8.**
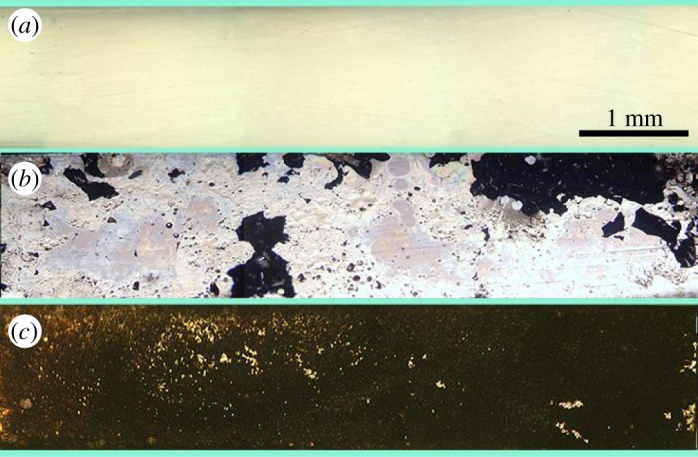
OM micrographs of electrode surfaces after tensile testing: (*a*) original electrode as a control; (*b*) electrode from the heat-adherence test and (*c*) electrode from the electrosurgical cutting test.

The electrode surfaces were examined by OM after tensile testing, and the typical morphologies are shown in figure 8. For the electrode sample from the electrosurgical cutting test, most of its surface is still covered with black sticking tissue after tensile testing, and the silver metal substrate of the electrode is exposed very locally (figure 8*c*). This means that the tensile failure is dominated by the intra-cohesional failure of sticking tissue. The actual critical tensile force, binding strength and energy dissipation of sticking tissue–electrode interface should be higher than those given in table 1. For the electrode sample from the heat-adherence test, the silver metal substrate is exposed on most of its surface after tensile testing (figure 8*b*), suggesting that the tensile failure is dominated by interface failure. The obtained binding strength is close to the actual binding strength of sticking tissue on electrode interface.

### 3.3. Tissue section and smoke analysis

**Figure 9.**
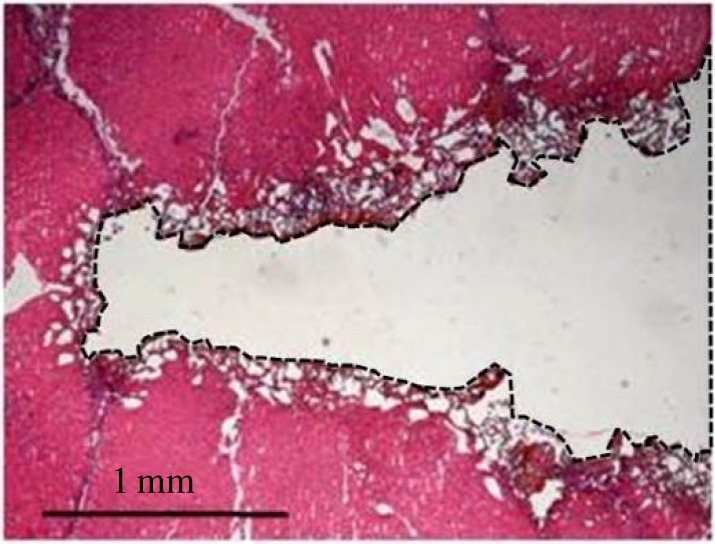
Histological examples of porcine liver tissue sample after the electrosurgical cutting test.

Figure 9 shows the histological example of incision in porcine liver tissue sample after the electrosurgical cutting test. Massive cavities appear in the porcine liver tissue around the incision, suggesting that some tissues near the incision are lost during the electrosurgical cutting.

**Figure 10.**
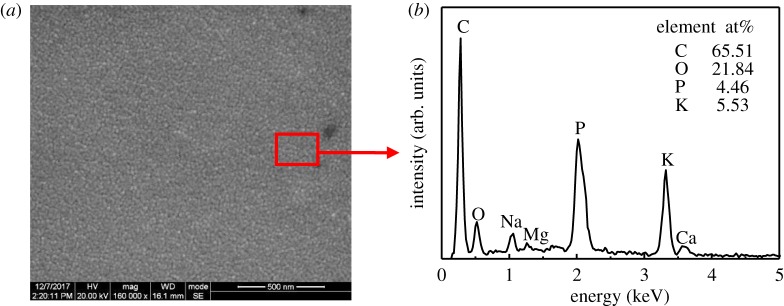
SEM micrograph and EDX spectrum of the electrode surface after smoke collection: (*a*) SEM micrograph and (*b*) EDX spectrum.

There is a great deal of smoke around the incision during electrosurgical cutting. An electrode heated and maintained at 350°C was put close to the incision during the electrosurgical cutting to collect the smoke. The collected smoke turns to carbonized fine particles with uniform size on the surface of electrode, as shown in figure 10. The size of these particles is comparable with the smallish particles of the sticking tissue from the electrosurgical cutting test (figure 6*a*), and their chemical composition is also similar.

## 4. Discussion

The aim of this study is to reveal the effect of high-frequency electric field on the tissue sticking of minimally invasive electrosurgical devices. Tissue sticking upon electrosurgical electrodes is a growing problem that troubles surgeons [7–12], while the mechanism governing the adhesion of tissue to electrodes is still unclear [23]. There simultaneously exist both thermal field and high-frequency electric field at the interface between the electrode and targeted tissue in electrosurgery, but little is known about the influence of electric field. To understand the effect of high-frequency electric field on the adhesion of tissue to electrodes, studying the tissue sticking just under heat would be necessary and helpful. In this study, the tissue sticking upon 304 stainless steel active electrode during electrosurgery has been investigated by comparing it with that just under thermal field which is referred to as the heat-adherence test.

Efforts were made to ensure these two test results were comparable. Firstly, porcine liver chunks and slices from the same *ex vivo* fresh porcine liver were used as tissue samples for these two tests, respectively. Secondly, the tissue temperature profile around the electrode was controlled to be approximately the same during these two tests, as shown in figure 2. Additionally, the test duration was also the same. As a result, both the starting tissue morphology and thermal energy input for these two tests are similar.

There is no significant difference in the chemical composition between the obtained sticking tissues from these two tests, as shown in figures 4 and 5. The 304 stainless steel electrode used in this study is mainly composed of Fe, Cr, Ni and Mn, and the porcine liver tissue mainly contains C, H, O, P and K, as well as a small amount of other elements. The sticking tissues are mainly composed of C, O, P and K with a little amount of Na, Mg and Ca. Their FTIR spectra are similar, but they are obviously different from that of the completely carbonized porcine liver tissue. And there exist no sugars in the sticking tissues. These observations suggest that the sticking tissues are the partially carbonized tissue debris, and no tissue sugaring occurs.

It is evident that the microstructures of the sticking tissues from these two tests are different, as well as their bonding with electrodes. As shown in figure 6, the sticking tissue after the electrosurgical cutting test consists of tightly packed nanoparticles with equable size and was firmly combined with the electrode. The binding strength of the sticking tissue on the electrode is more than 8.34 MPa (table 1 and figure 8). The sticking tissue from the heat-adherence test is composed of biggish nanoparticles with unequal size and had a loose and porous microstructure. Its binding strength with the electrode is only 0.53 MPa. Obviously, the compact sticking tissue from the electrosurgical cutting is much more difficult to be removed. Given that the sticking tissues from these two tests have similar chemical composition but different microstructure and bonding with the electrode, their formation mechanisms are different. That is to say, the sticking tissue from the electrosurgery procedure is not just as a result of tissue adherence and carbonization on the surface of hot electrode. The high-frequency electric field has an unignorable influence on tissue sticking.

**Figure 11.**
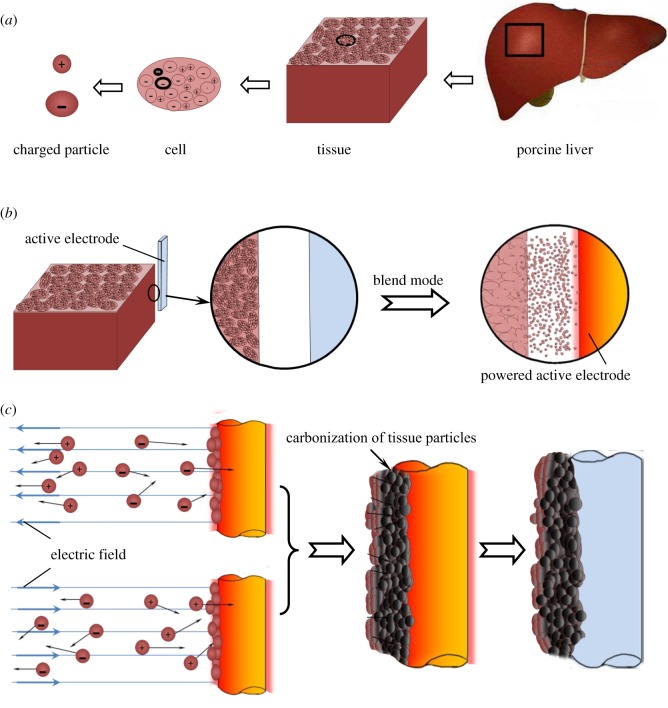
Schematic diagrams of porcine liver tissue, electrosurgery procedure and formation process of sticking tissue upon electrode: (*a*) porcine liver tissue; (*b*) electrosurgery procedure and (*c*) formation process of sticking tissue upon electrode.

Figure 11 illustrates the formation of sticking tissue upon active electrode during electrosurgery. Human tissue is made up of cells, and cellular cytoplasms contain charged particles or ions, as shown in figure 11*a*. The cations are from small atoms such as sodium, potassium and calcium, while the anions are from atoms such as chlorine and protein molecules (generally proteins exist in the form of negatively charged particles due to the alkaline ionization of amino acid in body) [4]. When an active electrode approaches porcine liver tissue during an electrosurgical procedure, high-frequency current is conducted to the targeted tissue to cause the wild oscillation of charged intracellular substances [4]. The wild oscillation of intracellular substances not only generates heat by friction to destroy their structures, but also produces a high shear rate to disintegrate the intracellular substances. As a result, the intracellular substances are fragmented to nanoparticles with uniform size, and the intracellular fluid is vaporized by heat. Finally, the cell explodes, and the nanoparticles burst out to form smoke (figure 11*b*). As shown in figures 9 and 10, the carbonized particles from the smoke are fine nanoparticles with uniform size, and there appear many reticular cavities beneath the incision due to the loss of cellular substance.

Owing to the existence of electric field, the charged particles in the smoke are accelerated and hit the hot electrode (about 350°C) (figure 11*c*). These particles are packed and carbonized on the electrode surface, forming the sticking tissue with a compact microstructure. The high-speed impact of charged particles roughens the surface of hot electrode (figure 7), resulting in very good bonding of sticking tissue with the electrode (table 1).

Compared with the electrosurgery, there is no electric field at all during the heat-adherence test. The charged intracellular substances do not oscillate without electric field, and the tissue directly adheres to the electrode. The cells are split under heat, and the cellular contents subsequently flow out. They are carbonized and contracted on the hot electrode surface, forming the sticking tissue. Because the intracellular substances are of various shape and size, the obtained sticking tissue has a loose and porous microstructure composed of nanoparticles with unequal size, as shown in figures 4 and 6. The surface morphology of electrode has no obvious change during this process, and the bonding of sticking tissue with the electrode is relatively weak (table 1).

In fact, there always exists high-frequency electric field during electrosurgery. It fragments the tissue to fine nanoparticles and accelerates them to impact against the electrode. Once surface texture is bigger than these nanoparticles, the anti-sticking effect of the hydrophobic texture on electrode surface would come down. Moreover, the high-speed impact of these particles on the electrode could weaken the anti-sticking of protective layers such as steam bubbles produced by cold saline irrigation, destroy the hydrophobic coating/surface texture on electrode surface and contribute to the high binding strength of sticking tissue with the electrode. This is why the current anti-sticking measures only alleviate tissue sticking to a certain degree, but cannot totally solve the problem. The results of the present study suggest that high-frequency electric field plays an extremely important role in the sticking tissue formation. It is the thermo-electric coupling that underlies the function of minimally invasive electrosurgical devices, and obviously its effect cannot be ignored in the tissue sticking study and anti-sticking strategies.

Although only one typical biological tissue was used in this study to investigate the tissue sticking upon electrodes under one electrosurgical mode, these results, to the best of our knowledge, represent a detailed analysis of the role of high-frequency electric field in the sticking tissue formation during electrosurgery. The finding of this study will extend the understanding of the adhesion of tissue to electrodes and provide valuable insights into the development of effective anti-sticking measures. And our future study will further explore the influence of high-frequency electric field on the interfacial bonding of sticking tissue and electrode.

## 5. Conclusion

In this study, the influence of high-frequency electric field on the tissue adhesion to electrodes during electrosurgery has been investigated by comparing it with that just under thermal field which is referred to as the heat-adherence test. Based on the given testing conditions, the conclusions are summarized as follows:

(1) Both the sticking tissues on the electrodes obtained from the electrosurgical cutting and the heat-adherence tests are partially carbonized porcine liver tissue, but there exist obvious differences in their microstructure and bonding with the electrode. The sticking tissue formed just under heat is composed of loose biggish nanoparticles with unequal size and has a weak bonding with the electrode, while the sticking tissue from the electrosurgical cutting test consists of tightly packed fine nanoparticles with equable size as a result of thermo-electric coupling and has a much stronger bonding with the electrode.
(2) High-frequency electric field plays an extremely important role in the formation of sticking tissue. It is the thermo-electric coupling that underlies the function of minimally invasive electrosurgical devices, and thus, the effect of high-frequency electric field cannot be ignored in the tissue sticking study and anti-sticking strategies.

## Supplementary Material

figures and table

## Data accessibility

The datasets supporting this article have been uploaded as part of the electronic supplementary material.
